# Supplementary material for: Perovskite-polymer composite cross-linker approach for highly-stable and efficient perovskite solar cells
Source: Nat Commun. 2019 Jan 31;10:520. doi: 10.1038/s41467-019-08455-z (PMC6355927; doi:10.1038/s41467-019-08455-z)
Supplement: Supplementary file 1 — Supplementary Information [file 41467_2019_8455_MOESM1_ESM.pdf]

SUPPLEMENTARY INFORMATION

Perovskite-Polymer Composite Cross-linker Approach for Highly-stable and Efficient Perovskite Solar Cells

Han et al.

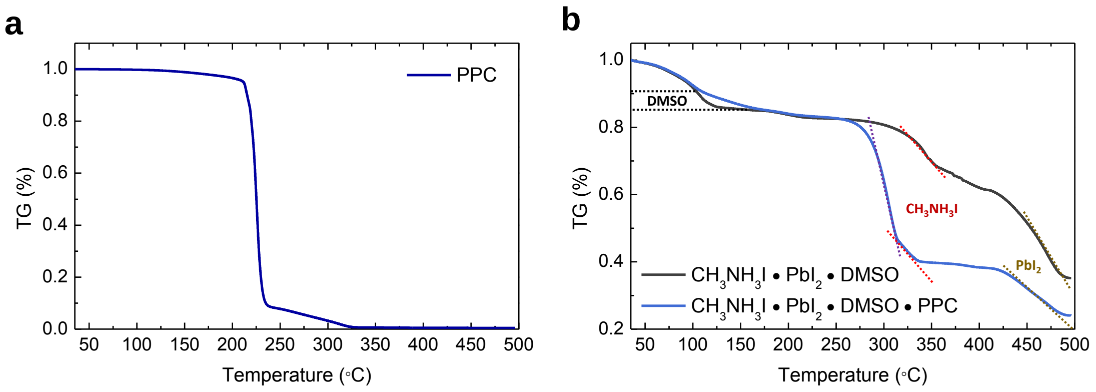

Supplementary Figure 1 | Thermogravimetric analysis (TGA). a, propylene carbonate (PPC) and **b**, adduct powders of $CH_3NH_3I{\cdot}PbI_2{\cdot}$ dimethyl sulfoxide (DMSO) and $CH_3NH_3I{\cdot}PbI_2{\cdot}DMSO{\cdot}PPC$.

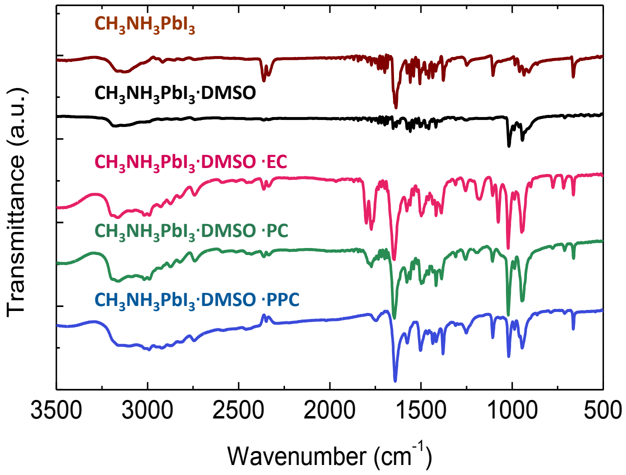

Supplementary Figure 2 | Fourier transform infrared (FTIR) spectra. FTIR of synthesized adduct powders of CH_3NH_3I and PbI_2 without and with Lewis bases.

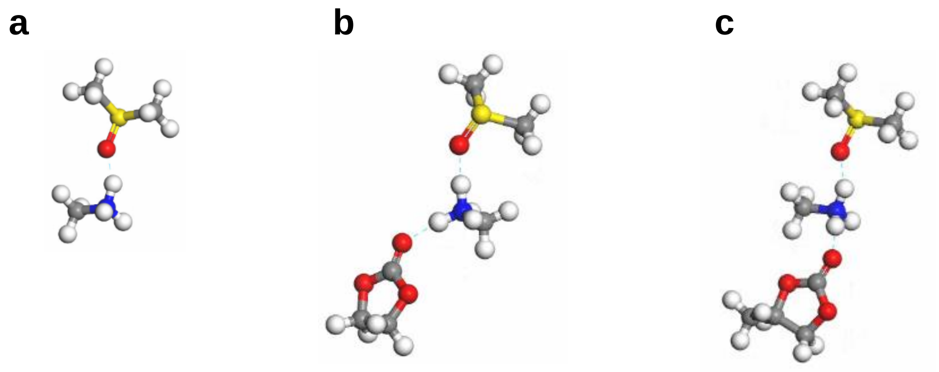

Supplementary Figure 3 | Most favorable molecular configurations. **a**, CH_3NH_3-DMSO, **b**, CH_3NH_3-DMSO-ethlyene carbonate (EC), and **c**, CH_3NH_3-DMSO-propylene carbonate (PC).

a b c d

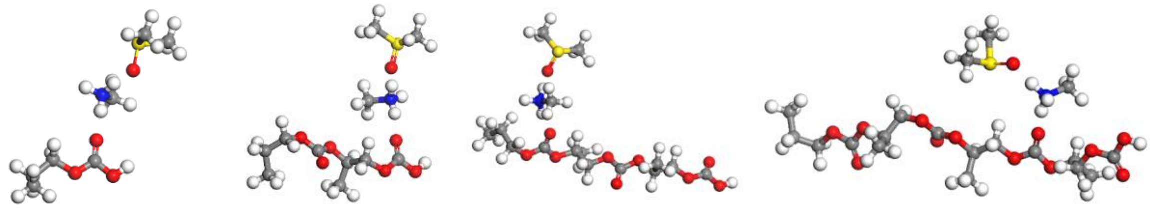

Supplementary Figure 4 | Most favorable molecular configurations according to the number of polymer repeating units. **a**, CH_3NH_3I-DMSO-$CH(CH_3)CH_2OCO_2$, **b**, CH_3NH_3I-DMSO-$[CH(CH_3)CH_2OCO_2]_2$, **c**, CH_3NH_3I-DMSO-$[CH(CH_3)CH_2OCO_2]_3$, **d**, CH_3NH_3I-DMSO-$[CH(CH_3)CH_2OCO_2]_4$

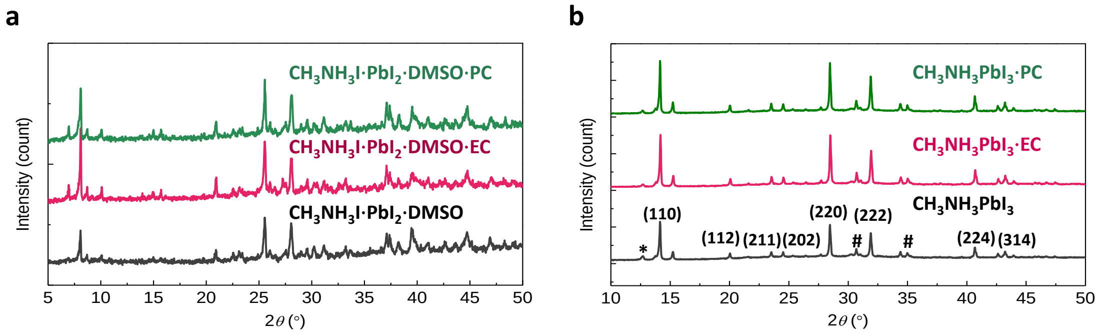

Supplementary Figure 5 | X-ray diffraction spectra of the synthesized adduct powders and perovskite films. a, X-ray diffraction spectra of the adduct powders of $CH_3NH_3I{\cdot}PbI_2{\cdot}DMSO$, $CH_3NH_3I{\cdot}PbI_2{\cdot}DMSO{\cdot}EC$, and $CH_3NH_3I{\cdot}PbI_2{\cdot}DMSO{\cdot}PC$ and of the **b**, perovskite films of $CH_3NH_3PbI_3$, $CH_3NH_3PbI_3{\cdot}EC$ and $CH_3NH_3PbI_3{\cdot}PC$.

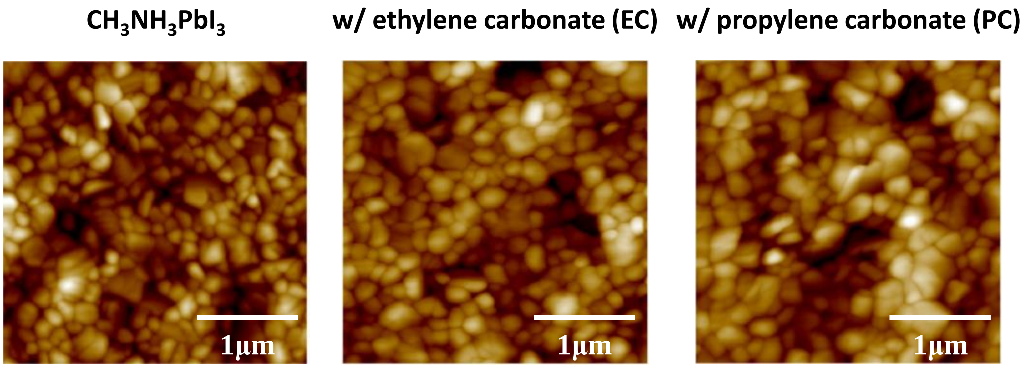

Supplementary Figure 6 | Surface morphology of the perovskite films. Atomic force microscopy images of **a**, $CH_3NH_3PbI_3$, **b**, $CH_3NH_3PbI_3$·EC and **c**, $CH_3NH_3PbI_3$·PC.

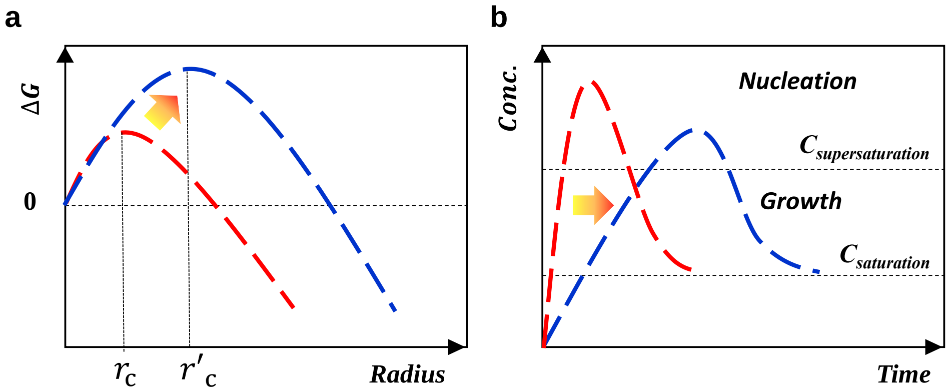

Supplementary Figure 7 | Perovskite crystallization kinetics. Schematic diagrams of **a**, free energy *vs*. cluster radius and **b**, nucleation and growth behaviours based on the LaMer model (red dashed line: small-molecular intermediate phase- and blue dashed line: macromolecular intermediate phase-induced crystallization).

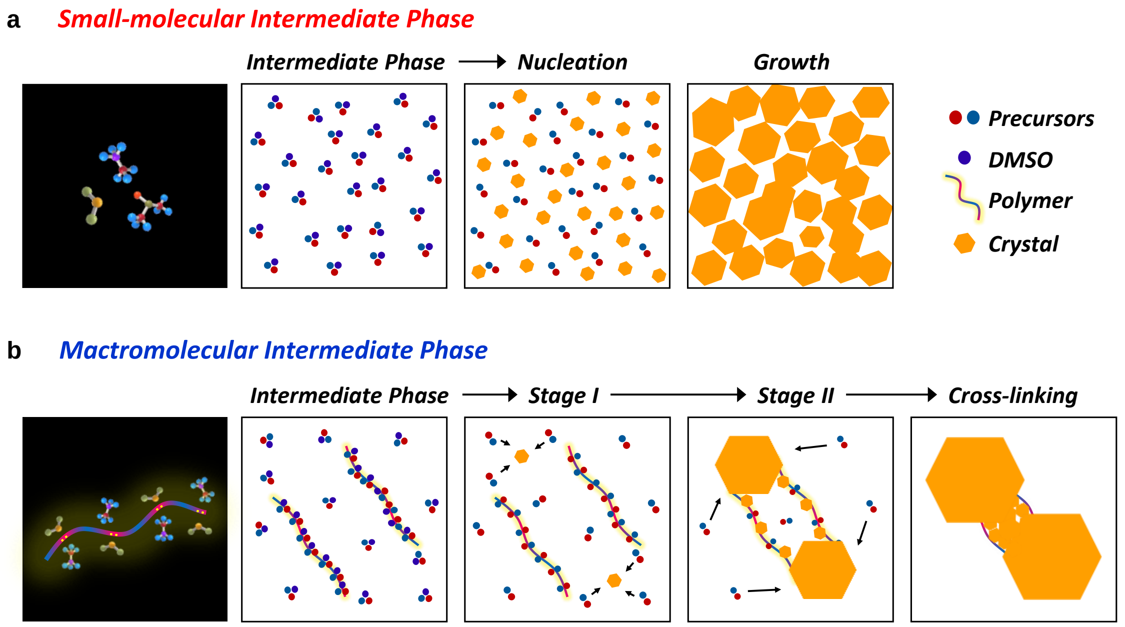

Supplementary Figure 8 | Crystallization mechanism. Schematic illustration of the nucleation and growth behavior of the **a**, small molecular intermediate phase- and **b**, macromolecular intermediate phase-induced crystallization and inter-grain cross-linking.

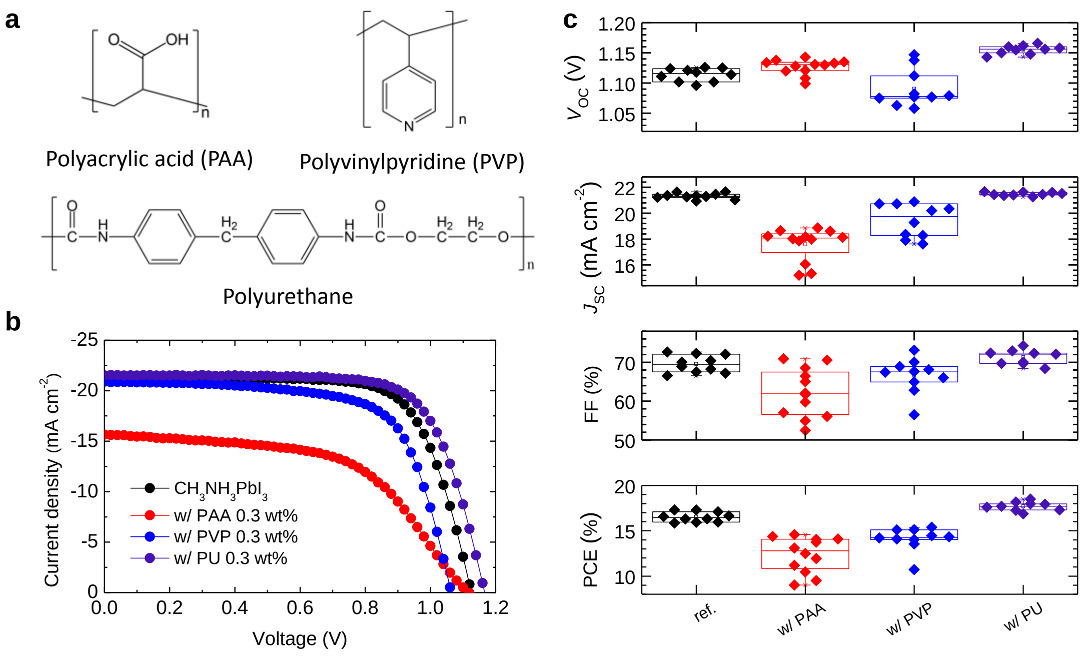

Supplementary Figure 9 | Photovoltaic performance of solar cells with different polymers. **a**, Chemical structure of polyacrylic acid (PAA), polyvinylpyridine (PVP), and polyurethane (PU), **b**, Current density versus voltage (*J*-*V*) characteristics of the best $CH_3NH_3PbI_3$ solar cells with various polymers, and **c**, photovoltaic parameters of $CH_3NH_3PbI_3$ perovskite solar cells with different kinds of polymers. (short-circuit current density (J_{SC}), open-circuit voltage (V_{OC}), fill factor (FF) and power conversion efficiency (PCE)). Each box chart includes the minimum, lower quartile (lower horizontal line), median (middle horizontal line), mean (hollow square), upper quartile (upper horizontal line), maximum, and discrete data.

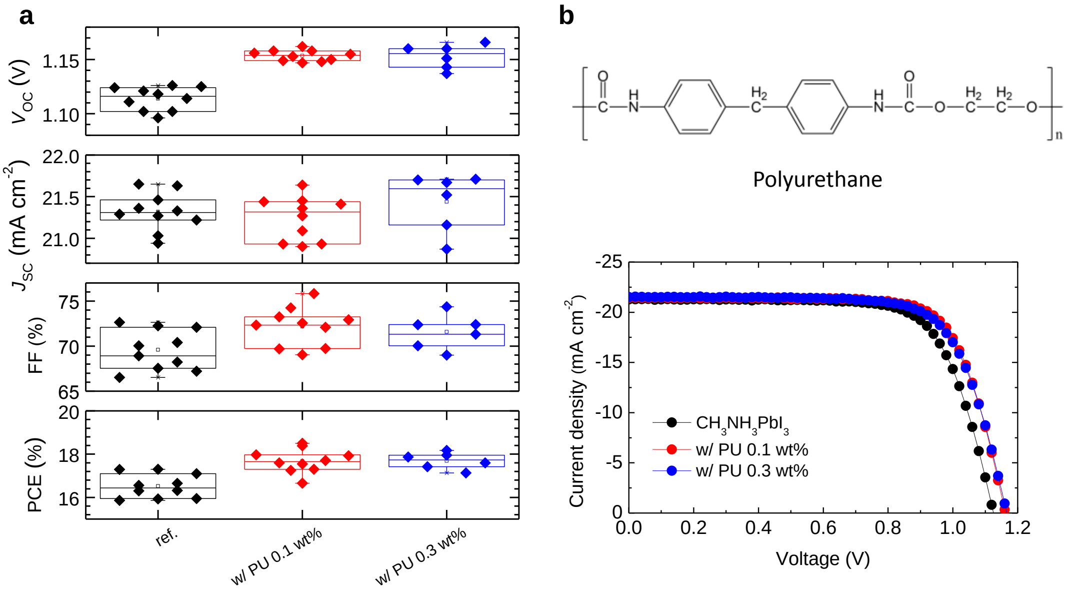

Supplementary Figure 10 | Photovoltaic performance of solar cells with polyurethane. a, photovoltaic parameters of $CH_3NH_3PbI_3$ perovskite solar cells as a function of concentration of PU added (short-circuit current density (J_{SC}), open-circuit voltage (V_{OC}), fill factor (FF) and power conversion efficiency (PCE)), each box chart includes the minimum, lower quartile (lower horizontal line), median (middle horizontal line), mean (hollow square), upper quartile (upper horizontal line), maximum, and discrete data, **b**, current density versus voltage (*J*-*V*) characteristics of the best $CH_3NH_3PbI_3$ solar cells with 0.1wt% and 0.3wt% PU.

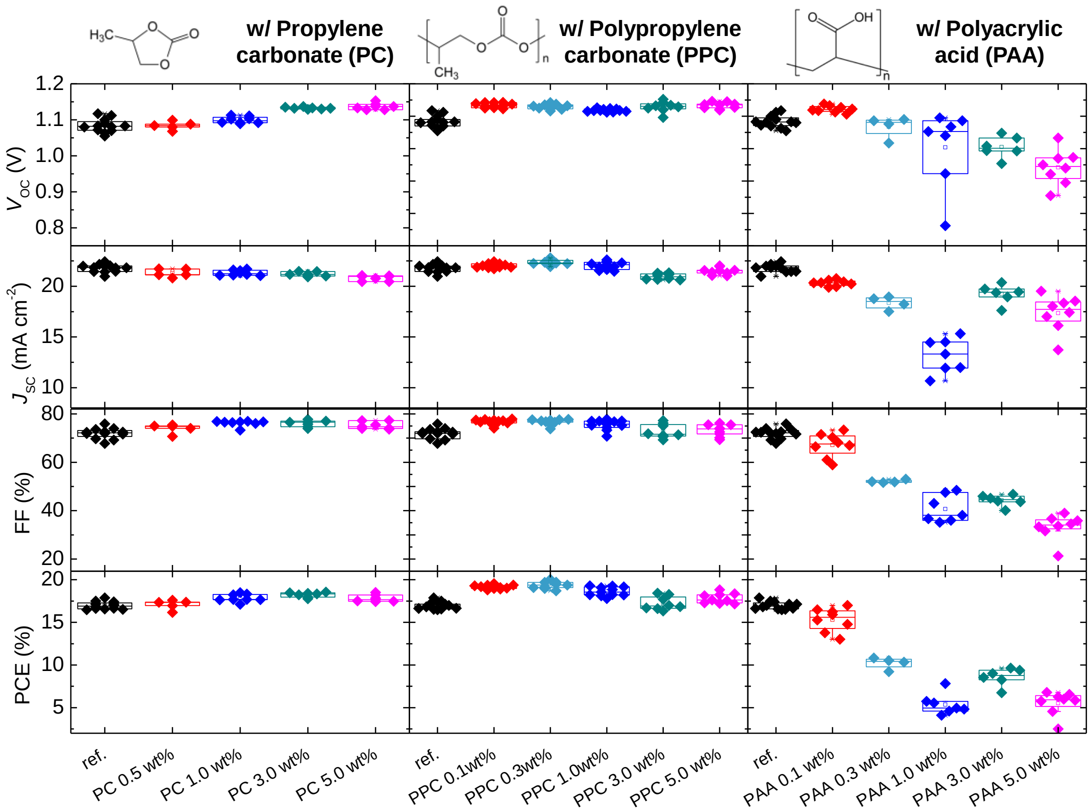

Supplementary Figure 11 | Photovoltaic performance of solar cells according to molecular concentration. Photovoltaic parameters of $CH_3NH_3PbI_3$ perovskite solar cells with PC, PPC, and PAA according to their molecular concentration. (short-circuit current density (J_{SC}), open-circuit voltage (V_{OC}), fill factor (FF) and power conversion efficiency (PCE)). Each box chart includes the minimum, lower quartile (lower horizontal line), median (middle horizontal line), mean (hollow square), upper quartile (upper horizontal line), maximum, and discrete data.

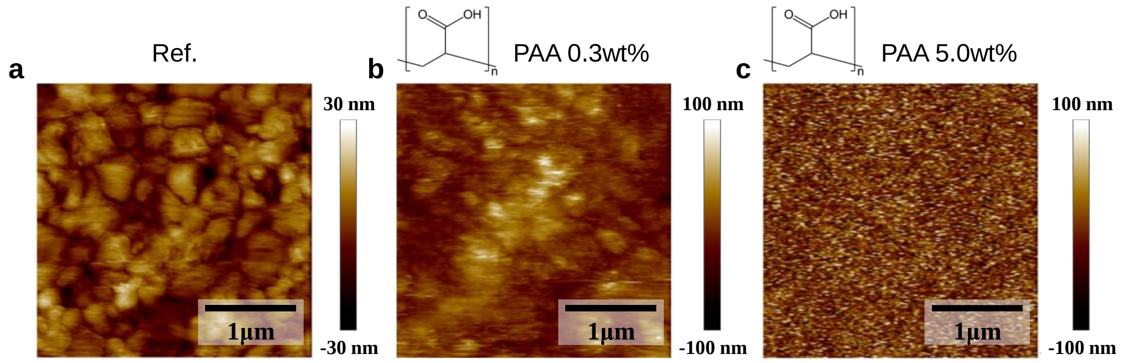

Supplementary Figure 12 | Surface Morphology of perovskite films with PAA. Atomic force microscopy images of $CH_3NH_3PbI_3$ film **a**, without, **b**, with 0.3wt% of PAA, and **c**, with 5.0wt% of PAA.

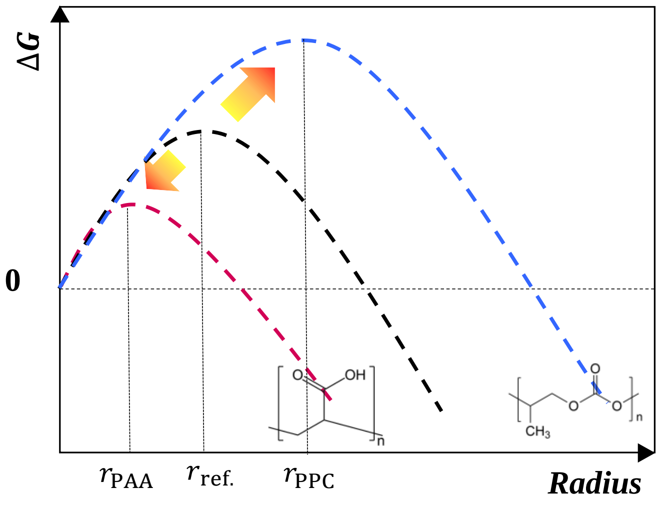

Supplementary Figure 13 | Schematic diagrams of free energy *vs.* cluster radius.

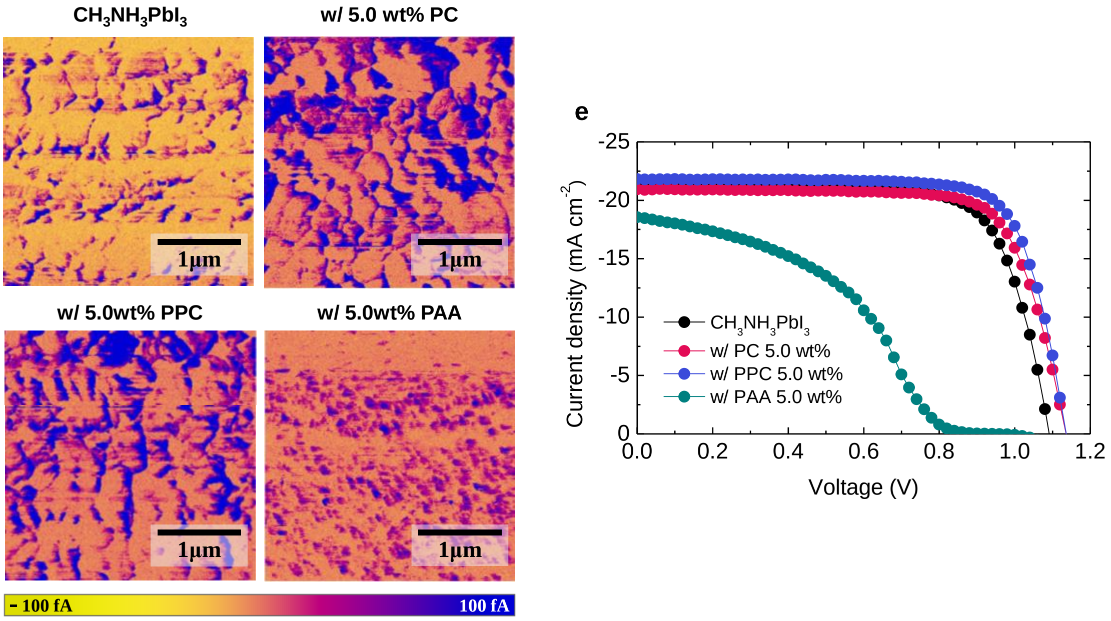

Supplementary Figure 14 | Electrical properties of perovskite films. Electrical current mapping measured by conductive atomic force microscopy of $CH_3NH_3PbI_3$ **a**, without, and with **b**, 5.0wt% of PC, **c**, 5.0wt% of PPC, **d**, 5.0wt% of PAA. **e**, current density *vs.* voltage (*J*-*V*) characteristics of perovskite solar cell without, and with 5.0wt% of PC, PPC, and PAA.

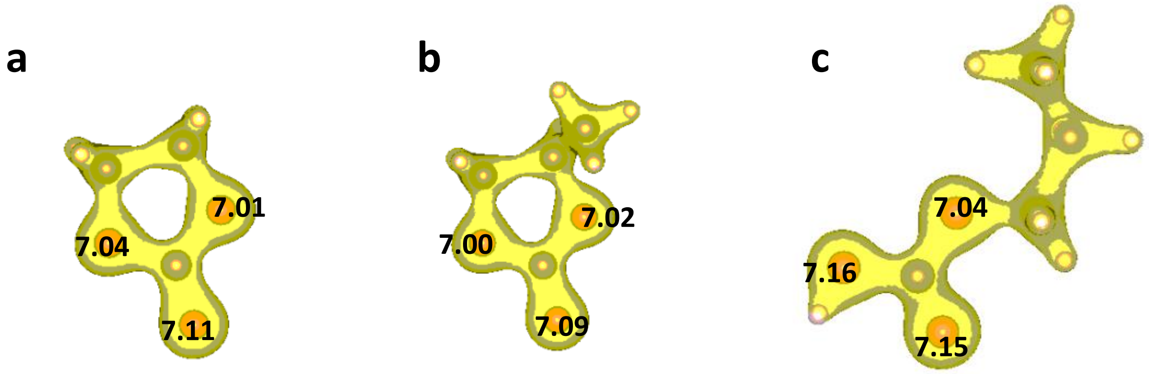

Supplementary Figure 15 | Isosurface of total charge densities. **a**, EC, **b**, PC and **c**, PPC (isosurface levels are 0.2 e bohr^{-3}) and Bader partial atomic charges of each oxygen atom are indicated in black.

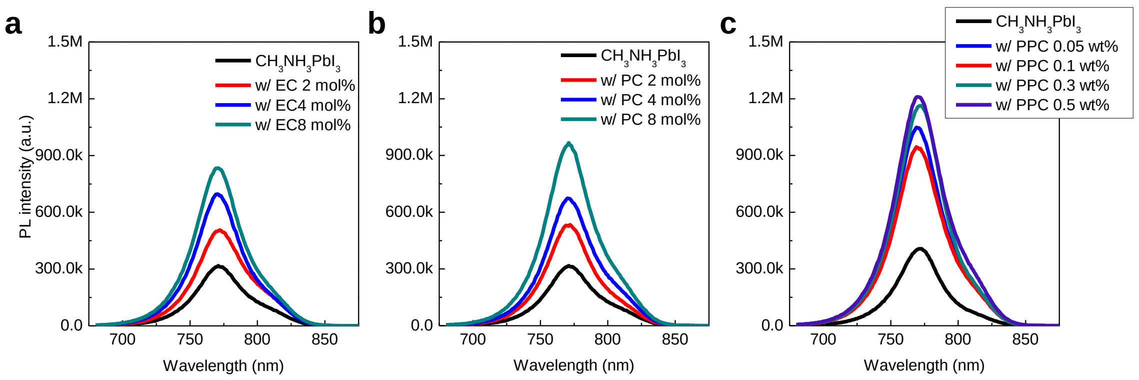

Supplementary Figure 16 | Photoluminescence spectra. PL intensity *vs.* wavelength of $CH_3NH_3PbI_3$ films with **a**, EC, **b**, PC and **c**, PPC added according to the concentration of additives.

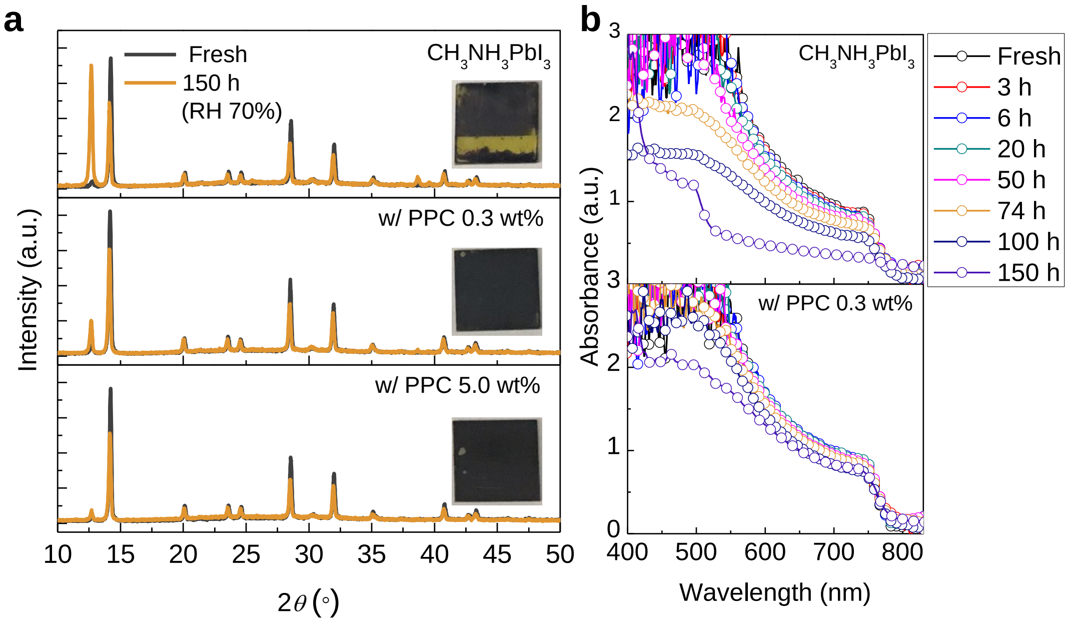

Supplementary Figure 17 | Stability of cross-linked perovskite film against moisture. a, X-ray diffraction spectra of bare $CH_3NH_3PbI_3$ and $CH_3NH_3PbI_3$·PPC films before (black) and after (yellow) 150 hours exposure to moisture (relative humidity: 70±5%), **b**, Absorption spectra of bare $CH_3NH_3PbI_3$, and $CH_3NH_3PbI_3$·PPC films according to their exposure time to moisture.

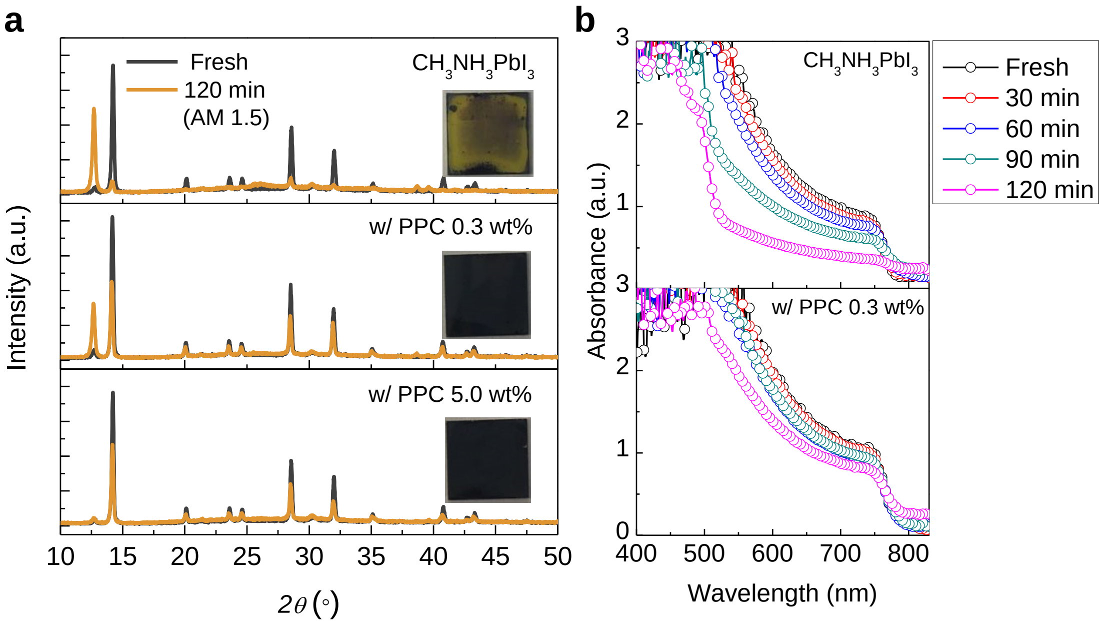

Supplementary Figure 18 | Stability of cross-linked perovskite film against light illumination. a, X-ray diffraction spectra of bare $CH_3NH_3PbI_3$ and $CH_3NH_3PbI_3$·PPC (0.3 and 5.0 wt%) films before (black) and after (yellow) 120 min illumination of light (1.5 AM), **b**, absorption spectra of bare $CH_3NH_3PbI_3$, and $CH_3NH_3PbI_3$·PPC films according to exposure time to light illumination.

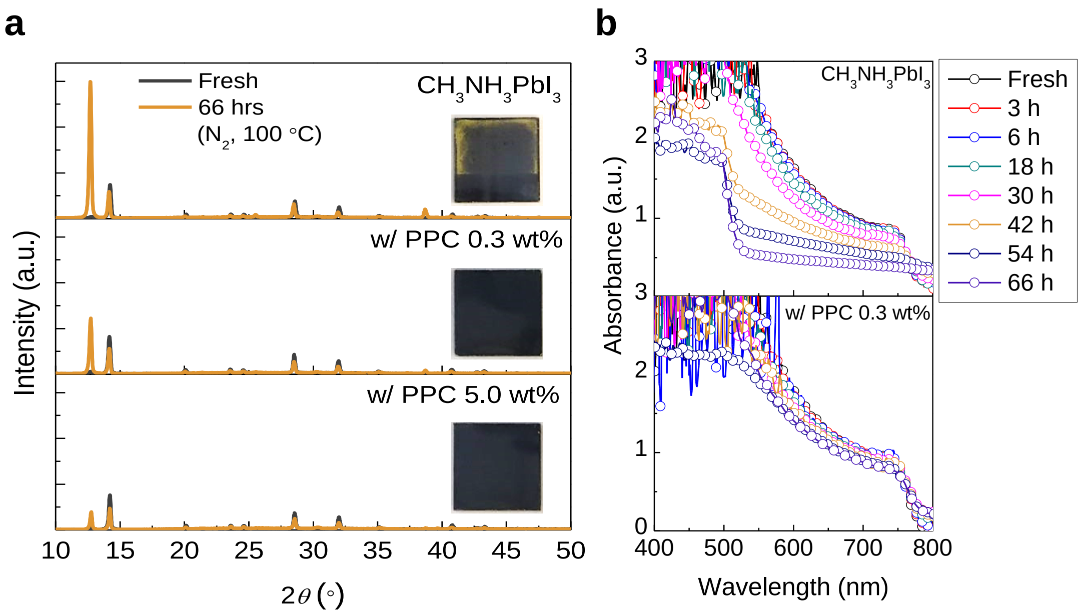

Supplementary Figure 19 | Stability of the cross-linked perovskite films against high temperature. a, X-ray diffraction spectra of bare $CH_3NH_3PbI_3$ and $CH_3NH_3PbI_3$·PPC (0.3 and 5.0 wt%) films before (black) and after (yellow) 66 hours of heating at 100 ℃ in nitrogen atmosphere, **b**, absorption spectra of bare $CH_3NH_3PbI_3$, and $CH_3NH_3PbI_3$·PPC films according to heating time.

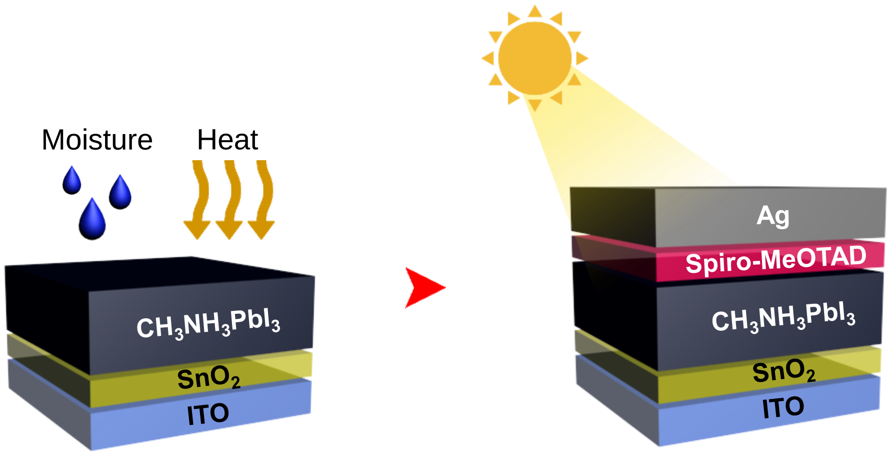

Supplementary Figure 20 | Schematic illustration of the environmental stability test on the perovskite solar cells

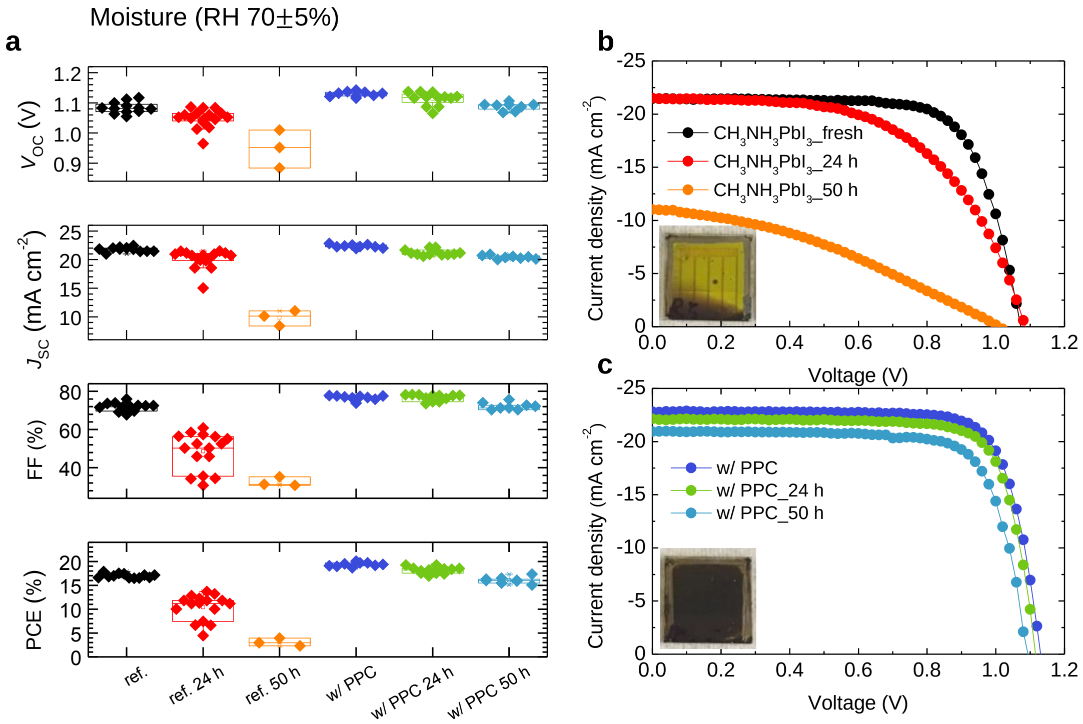

Supplementary Figure 21 | Moisture stability of solar cells. a, Photovoltaic parameters of $CH_3NH_3PbI_3$ perovskite solar cells according to exposure time against moisture (RH 70±5%) without/ with PPC (short-circuit current density (J_{SC}), open-circuit voltage (V_{OC}), fill factor (FF) and power conversion efficiency (PCE)), Each box chart includes the minimum, lower quartile (lower horizontal line), median (middle horizontal line), mean (hollow square), upper quartile (upper horizontal line), maximum, and discrete data, Current density versus voltage (*J*-*V*) characteristics of the best $CH_3NH_3PbI_3$ solar cells as a function of exposure time **b**, without, and **c**, with PPC.

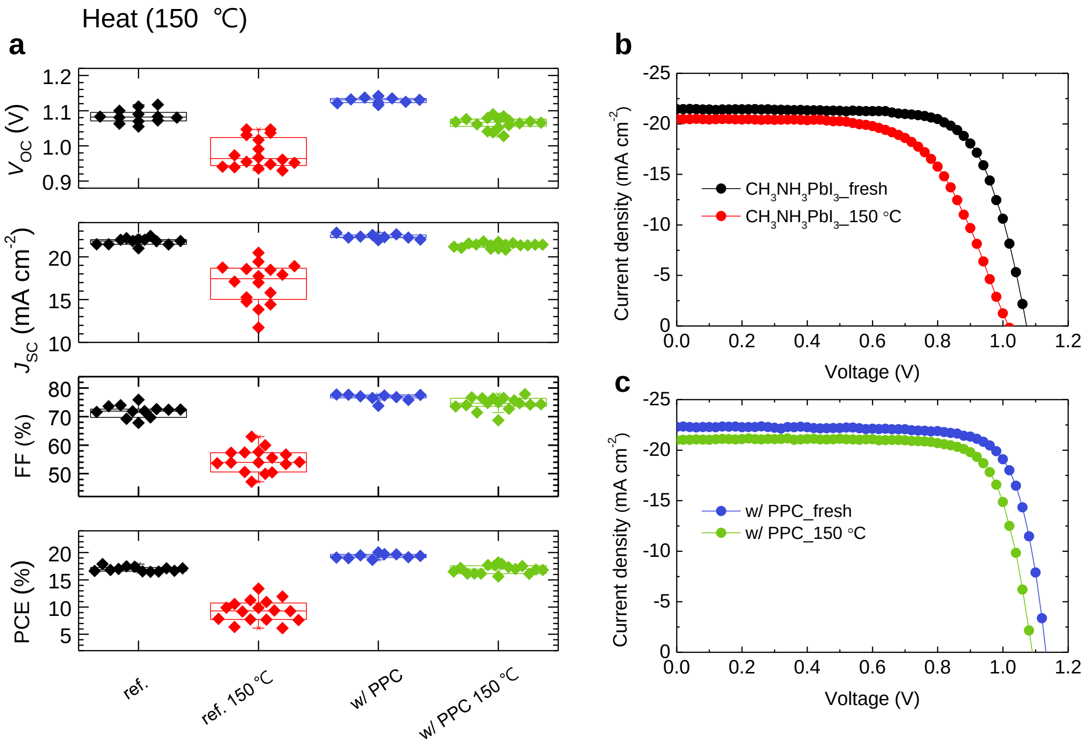

Supplementary Figure 22 | Thermal stability of solar cells. a, Photovoltaic parameters of $CH_3NH_3PbI_3$ perovskite solar cells according to exposure against high temperature (150 ℃) without/ with PPC (short-circuit current density (J_{SC}), open-circuit voltage (V_{OC}), fill factor (FF) and power conversion efficiency (PCE)), Each box chart includes the minimum, lower quartile (lower horizontal line), median (middle horizontal line), mean (hollow square), upper quartile (upper horizontal line), maximum, and discrete data, Current density versus voltage (*J*-*V*) characteristics of the best $CH_3NH_3PbI_3$ solar cells **b**, without, and **c**, with PPC.

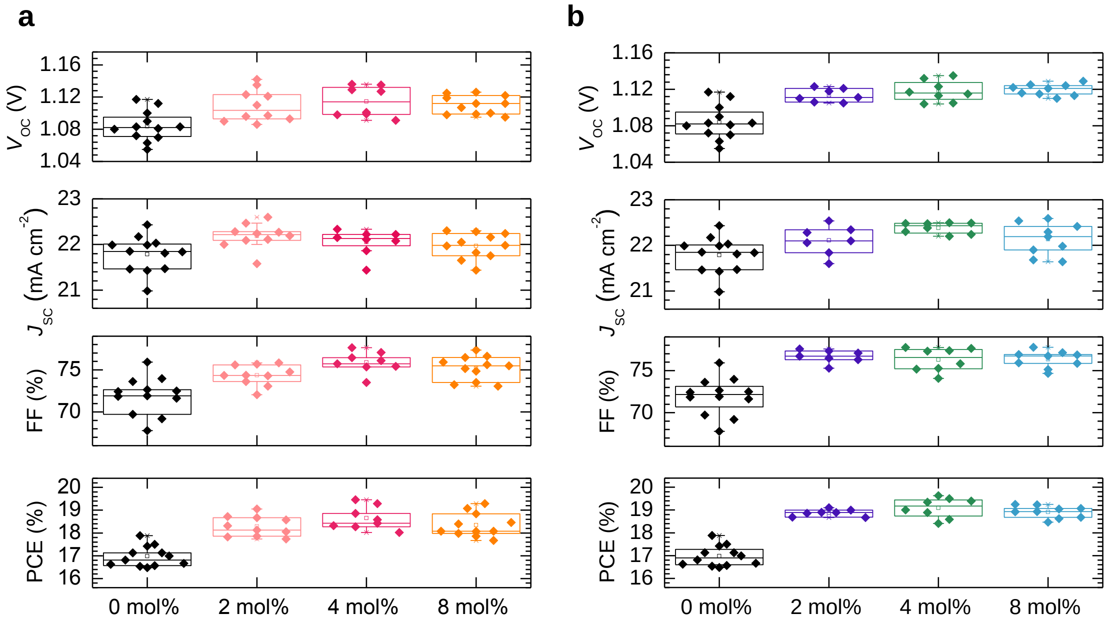

Supplementary Figure 23 | Photovoltaic performance of $CH_3NH_3PbI_3$ perovskite solar cells with small molecular Lewis bases. Photovoltaic parameters of $CH_3NH_3PbI_3$ perovskite solar cells as a function of the amount of **a**, EC and **b**, PC added: short-circuit current density (J_{SC}), open-circuit voltage (V_{OC}), fill factor (FF) and power conversion efficiency (PCE). Each box chart includes minimum, lower quartile (lower horizontal line), median (middle horizontal line), mean (hollow square), upper quartile (upper horizontal line), maximum, and discrete data.

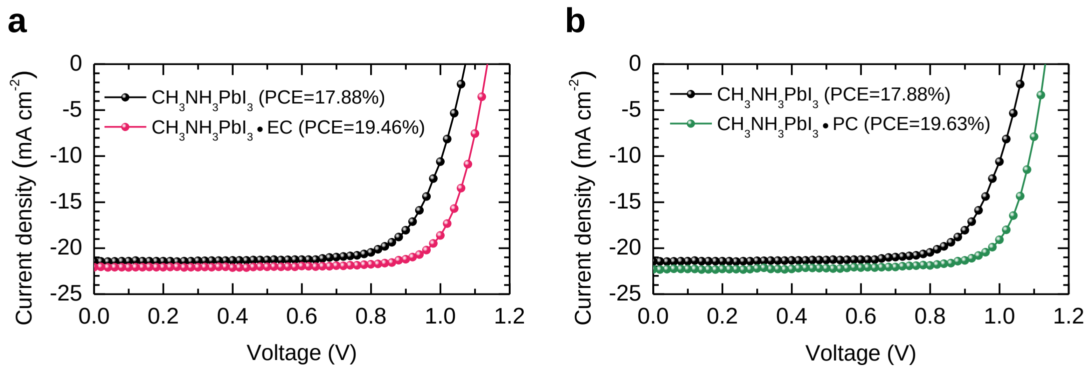

Supplementary Figure 24 | Best-performing $CH_3NH_3PbI_3$ perovskite solar cells with EC and PC. Current density versus voltage (*J*-*V*) characteristics of the best $CH_3NH_3PbI_3$ solar cells with **a**, EC and **b**, PC added.

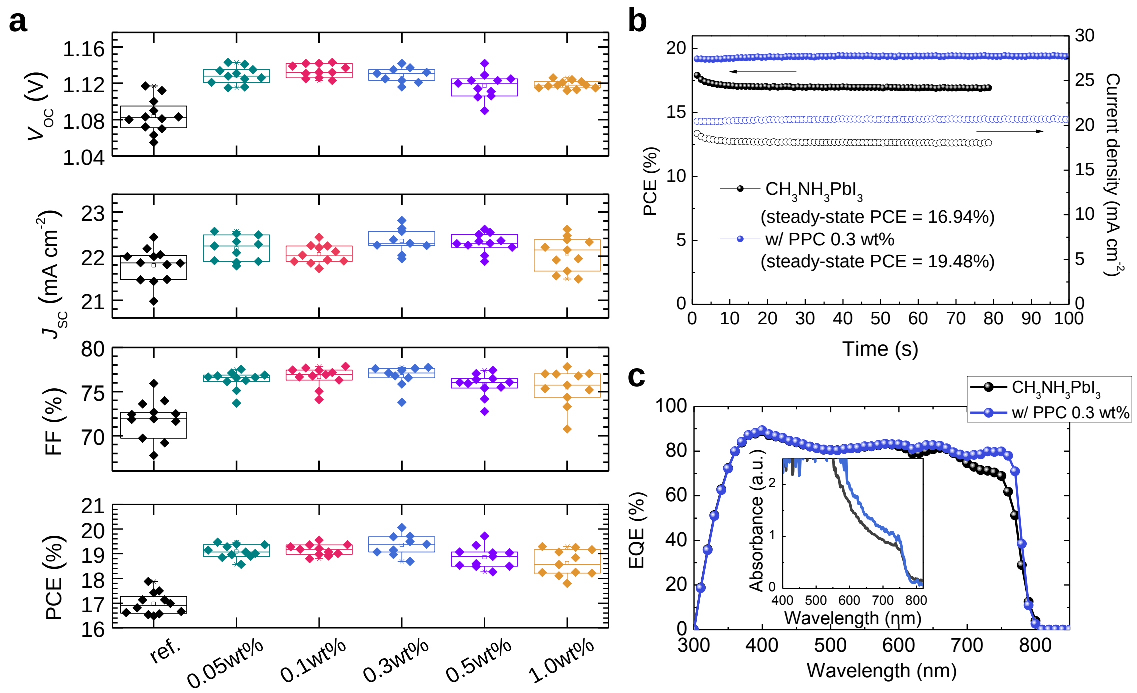

Supplementary Figure 25 | Photovoltaic performance of $CH_3NH_3PbI_3$ perovskite solar cells with PPC. a, Photovoltaic parameters of $CH_3NH_3PbI_3$ perovskite solar cells as a function of amount of PPC added: short-circuit current density (J_{SC}), open-circuit voltage (V_{OC}), fill factor (FF) and power conversion efficiency (PCE), Each box chart includes minimum, lower quartile (lower horizontal line), median (middle horizontal line), mean (hollow square), upper quartile (upper horizontal line), maximum, and discrete data. **b**, Steady-state PCE and **c**, external quantum efficiency (EQE) spectra of $CH_3NH_3PbI_3$ perovskite solar cells without and with PPC added.

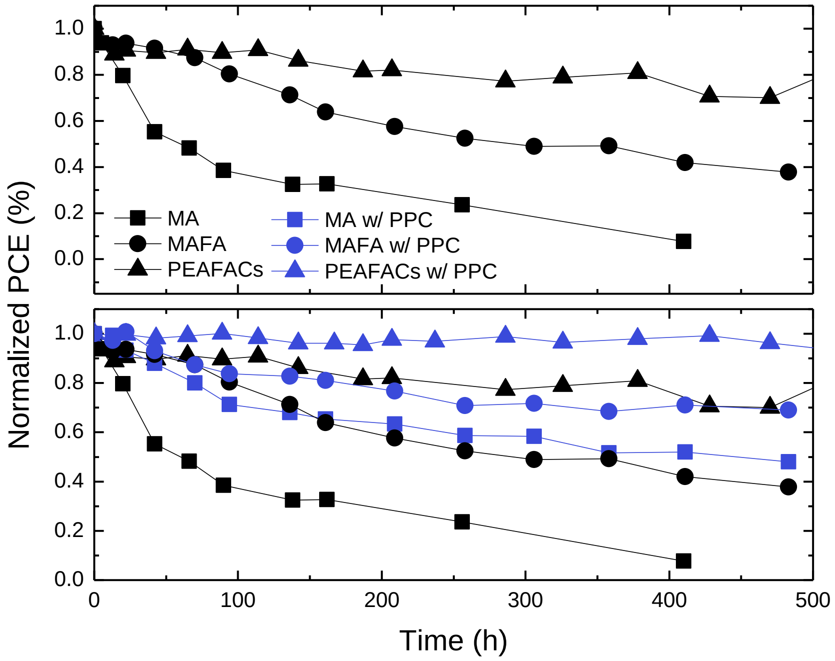

Supplementary Figure 26 | Operational stability of solar cells. PCE evolution under light illumination (1.5 AM) as a function of time of encapsulated solar cells without and with the polymeric Lewis base (PPC).

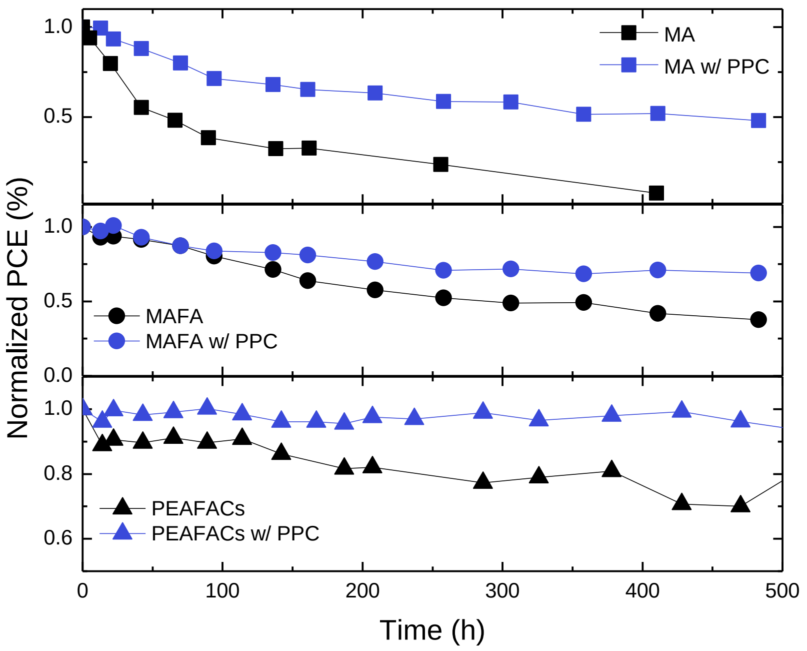

Supplementary Figure 27 | Operational stability of solar cells. PCE evolution under light illumination (1.5 AM) as a function of time of encapsulated solar cells without and with the polymeric Lewis base (PPC).

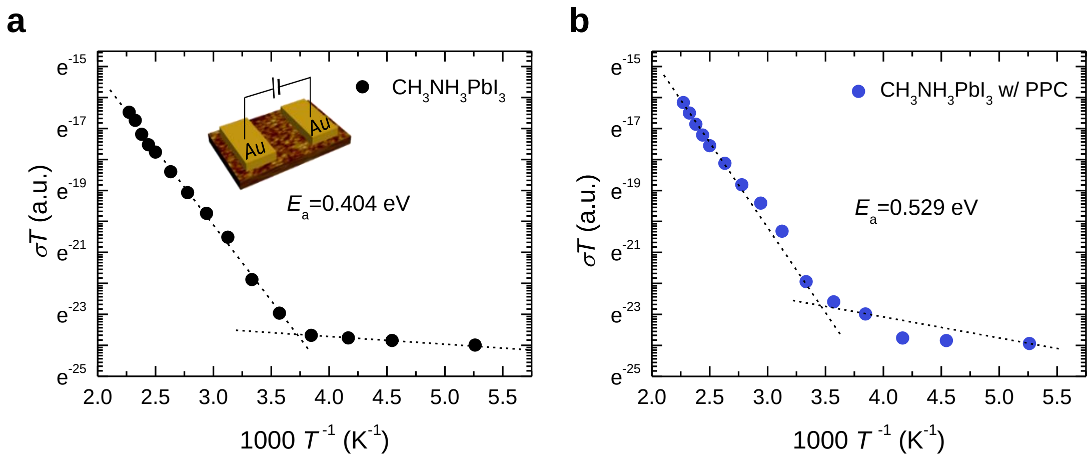

Supplementary Figure 28 | Activation energy for ion migration. Temperature-dependent conductivity of $CH_3NH_3PbI_3$ film **a**, without and **b**, with PPC (inset: schematic illustration of the lateral conduction device configuration.)

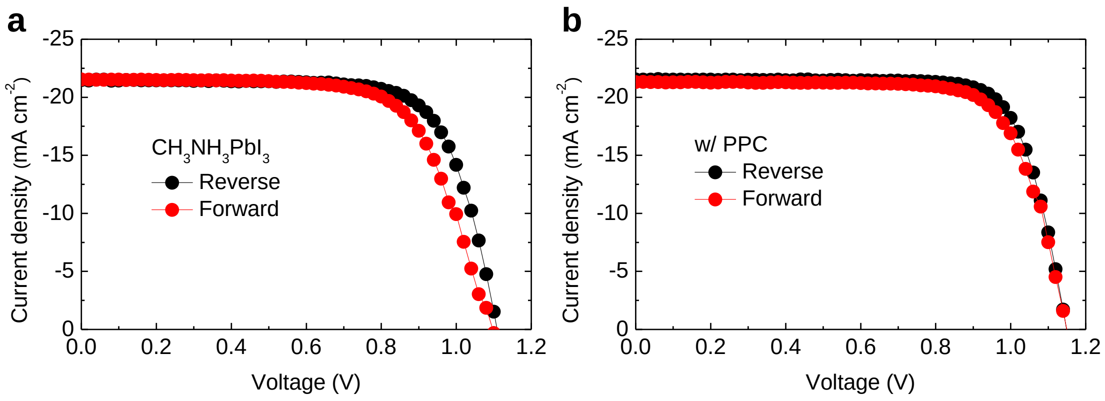

Supplementary Figure 29 | *J-V* hysteresis of solar cells. Current density versus voltage (J-V) characteristics of the $CH_3NH_3PbI_3$ solar cells according to scanning direction **a**, without, and **b**, with PPC. (Reverse: 1.2 V to -0.1V, Forward: -0.1 V to 1.2 V).

Supplementary Table 1 | Calculated intermolecular interaction energies of Lewis-acid-base adducts.

Adduct	Interaction Energy (eV)
$CH_3NH_3 \cdot DMSO$	-1.345
$CH_3NH_3 \cdot DMSO \cdot EC$	-2.225
$CH_3NH_3 \cdot DMSO \cdot PC$	-2.256

Supplementary Table 2 | Calculated intermolecular interaction energies of the Lewis acid-base adducts.

**Adduct**	**Interaction Energy (eV)**
$CH_3NH_3 \cdot DMSO$	-1.345
$CH_3NH_3 \cdot DMSO \cdot CH(CH_3)CH_2OCO_2$	-2.111
$CH_3NH_3 \cdot DMSO \cdot [CH(CH_3)CH_2OCO_2]_2$	-2.076
$CH_3NH_3 \cdot DMSO \cdot [CH(CH_3)CH_2OCO_2]_3$	-2.182
$CH_3NH_3 \cdot DMSO \cdot [CH(CH_3)CH_2OCO_2]_4$	-2.283

Supplementary Table 3 | Calculated intermolecular interaction energies of Lewis acid-base adducts.

	EC	PC	PPC
**Calculated dipole moment**	5.42 D	5.57 D	4.64 D
**Average partial atomic charge on oxygens**	7.05 e	7.04 e	7.12 e
**Charge transfer**	0.008 e	0.014 e	0.030 e

Supplementary Table 4 | Initial decay and calculated T_{80} of solar cells under light illumination.

**'A'-site cations**	**Initial decay [%]**	**T_{80} [h]**
MA	44.7	169.5
MA w/ PPC	29.5	391.0
MAFA	36.0	288.0
MAFA w/ PPC	16.2	929.6
FACs	10.4	420.8
FACs w/ PPC	1.8	10800.1

Supplementary Note 1

Thermogravimetric analysis (TGA)

Thermogravimetric analysis (TGA) of the PPC and synthesized adduct powders were conducted to investigate thermal properties and mass loss behaviors of polymeric Lewis base and their intermediate phase (Supplementary Figure 1). PPC is thermally stable and thermal decomposition of PPC was not observed at temperaure below 200 ℃, whereas DMSO was found to be volatile at temperature around 70 ℃[1]. After around 10% weight loss that indicates sublimation of DMSO at 70 ℃, TGA of $CH_3NH_3I{\cdot}PbI_2{\cdot}DMSO$ adduct showed two distint weight loss steps at 320 ℃ and 450 ℃ of which weight losses indicate sublimation of CH_3NH_3I and thermal decomposition of PbI_2, respectively[2]. PPC undergoes 100% weight loss in one step of which the onset is at 225 ℃ suggesting sublimation of PPC, but its adduct powder with perovskite precursors (*i.e.*, $CH_3NH_3I{\cdot}PbI_2{\cdot}DMSO{\cdot}PPC$) exhibited sharp weight loss transition at 300 ℃ that is higher than that of pure PPC. Weight loss at 300 ℃ in $CH_3NH_3I{\cdot}PbI_2{\cdot}DMSO{\cdot}PPC$ can be speculated as sublimation of $CH_3NH_3I{\cdot}PbI_2{\cdot}PPC$ becuase two individual amounts of weight loss steps of CH_3NH_3I (320 ℃) and PbI_2 (450 ℃) were obviously decreased in the polymer adduct, which means that a long chain polymeric Lewis base strongly interact with perovskite precursors forming the macromolecular adduct.

Supplementary Note 2

Performance comparison depending on different functional groups

Three kinds of commercially available polymers, polyacrylic acid (PAA), poly(4-vinylpyridine) (PVP), and polyurethane (PU) were used for the comparison (Supplementary Figure 9a). All the polymers have lone pairs of electrons on nitrogen or oxygen along the polymer backbone, but different basicity and molecular dipole moments. Pyridine (C_5H_5N) and urea (CH_4N_2O) are the Lewis base functional units of PVP and PU, respectively, but the dipole moment of urea (4.56 D) is higher than that of pyridine (2.2 D). Compared to the reference device ($CH_3NH_3PbI_3$ without polymeric additives) fabricated in the same batch, the addition of PAA and PVP into the $CH_3NH_3PbI_3$ perovskite degraded its photovoltaic performance, while a small amount of PU increased the photovoltaic performance of the perovskite solar cells. The addition of PU mainly improved the open circuit voltage and fill factor, increasing the power conversion efficiency (PCE) of the $CH_3NH_3PbI_3$ perovskite solar cells, similar to the effect of adding a PPC.

Supplementary Note 3

Crystal growth with different molecules

The different effects of the small molecular Lewis base (*i.e.*, PC), polymeric Lewis base (*i.e.*, PPC), and polymeric acid (*i.e.*, PAA) on the electrical properties of the films and subsequent photovoltaic performances of the devices were investigated. We examined the photovoltaic performances of the perovskite solar cells according to the molecular concentrations ranging from 0.1wt% to 5.0wt%. We observed that the small molecule (PC) and polymeric Lewis base (PPC) mainly improved the open circuit voltages (V_{OC}) and fill factors (FF) of the devices, which showed no noticeable decrease even at high concentrations (Supplementary Figure 11). In contrast, PAA gradually decreased the grain size with increasing amounts of PAA added (Supplementary Figure 12). All the $CH_3NH_3PbI_3$ grains were finely cleaved into small-sized grains upon the addition of 5.0wt% of PAA (Supplementary Figure 12c), which could be attributed to the chemical interaction between PAA and the perovskite precursors/ or solvent in the solution. Per classical theory for homogeneous nucleation, the nucleation rate is described by using a critical free energy (ΔG_c), which represents the free energy required for nuclei to be stable without being dissolved in the solution and is the sum of the surface and bulk free energy of the nuclei. This critical free energy is defined as the activation energy for nucleation and used to describe the nucleation rate using an Arrhenius type equation[3]:

$$\frac{\mathrm{d}N}{\mathrm{d}t} = A\exp\left(-\frac{\Delta G_\mathrm{c}}{k_\mathrm{B}T}\right) \quad (1)$$

where *t* is time, *N* is number of nuclei, *A* is pre-exponential factor, k_B is Boltzmann`s constant, *T* is temperature. The crystal free energy (ΔG_c) can be written as a function of surface energy γ, molar volume *v*, supersaturation of solution *S*, which produce a following equation,

$$\frac{\mathrm{d}N}{\mathrm{d}t} = A\exp\left(-\frac{16\pi\gamma^3 v^2}{3k_\mathrm{B}^3T^3(\ln S)^2}\right) \quad (2)$$

Because the repeating functional group of PAA is a carboxylic acid (C_2H_3COOH) (pK_a= 4.25), the PAA likely interacted with the Lewis base solvents of the precursor solution (i.e., DMSO, and DMF), instead of forming an adduct with the Lewis acidic precursors, which possibly increased the saturation level of the precursors in the solution, resulting in a decreased activation energy for crystallization (Supplementary Figure 13). As a result, the fast crystallization formed a large number of nuclei, and subsequently small crystal grains, which could severely interrupt the inter-grain electrical coupling due to the insulating nature of PAA residing in between the small perovskite crystals.

Supplementary Note 4

Electrical properties of perovksite films with different molecules

The perovskite films were deposited on an ITO/ SnO_2 (electron transporting layer). To analyze their charge carrier conducting characteristics, a positive bias was applied using a Sb-doped Si tip. Both the small molecular Lewis base and polymeric Lewis base resulted in the overall electrical enhancement of the perovskite films compared to that of the bare $CH_3NH_3PbI_3$ film. Particularly, the electrical conductivity in the grain boundaries was seen to significantly increase even though the film had high concentrations of the insulating organic molecules (5.0wt%). The conductivity enhancements could be attributed to an enhanced charge carrier mobility due to the higher crystallinity and an increased charge carrier concentration due to a reduction in both the defect trapping sites and non-radiative recombination in the perovskite grains. In contrast, the $CH_3NH_3PbI_3$ film with 5.0wt% PAA incorporated exhibited electrically decoupled tiny grains and inhomogeneity in between the perovskite grains (Supplementary Figure 14). As a result, the existence of the insulating PAA in the perovskite film severely disrupted the electrical properties of the solar cells, degrading its J_{SC} and FF significantly (Supplementary Figure 14e).

Supplementary Note 5

Environmental stability of solar cells

After the perovskite layers were exposed to a humid environment (RH 70 ± 5%) (Supplementary Figure 20), the photovoltaic performances of the bare $CH_3NH_3PbI_3$ solar cells dropped significantly, and the working devices exhibited very poor performances, mainly having huge drops in J_{SC} and FF (50 h exposed bare $CH_3NH_3PbI_3$ device: V_{OC}: 0.949±0.06 V, J_{SC}: 9.85±1.34 mA cm^{-2}, FF: 0.325±2.4, and PCE: 3.08±0.81%) (Supplementary Figure 21). In contrast, the cross-linked $CH_3NH_3PbI_3$ with PPC showed much higher moisture resistance and still maintained relatively high photovoltaic performances even after 50 h exposure in the high moisture environment (50 h exposed PPC- $CH_3NH_3PbI_3$ device: V_{OC}= 1.088±0.02 V, J_{SC}: 20.43±0.28 mA cm^{-2}, FF: 0.723±1.8, and PCE: 16.07±0.67%). The high temperature heating of the bare $CH_3NH_3PbI_3$ also caused the photovoltaic performances of the devices to drop significantly (V_{OC}: 0.980 ± 0.04 V, J_{SC}: 16.89 ± 2.37 mA cm^{-2}, FF: 0.547 ± 4.0, PCE: 9.31 ± 2.02%) (Supplementary Figure 22). Compared to bare $CH_3NH_3PbI_3$, the PPC-$CH_3NH_3PbI_3$ cross-linked devices did not show any significant drop even after heated at 150 ℃ (V_{OC}: 1.064±0.07 V, J_{SC}: 21.34±0.28 mA cm^{-2}, FF: 0.746±2.3, PCE: 16.93±0.77%), and proved their better thermal stress tolerance as seen in the XRD and UV-vis-Abs results.

Supplementary Note 6
Ion migration in perovskite films

The Nernst-Einstein relation ($\sigma(T) = \frac{\sigma_0}{T}\exp(\frac{-E_a}{kT})$, where k is the Boltzmann constant and σ_0 is a constant) was used to calculate the activation energy for ion migration, and the lateral conduction configuration was employed for the measurement (Supplementary Figure 28 inset). Our macromolecular adduct approach, which resultantly modified the perovskite crystallization, effectively increased the ion migration activation energy from 0.40 eV (bare $CH_3NH_3PbI_3$) to 0.53 eV ($CH_3NH_3PbI_3$ with PPC) (Supplementary Figure 28), which can be attributed to the reduction of the perovskite's charged defects (*e.g.*, positively charged anion vacancies) at the grain boundaries due to the passivation effect of the polymeric Lewis bases that remained in between the perovskite grains, and this translated to a reduced *J*-*V* hysteresis of the solar cells with PPC.

Supplementary References

1. Lee, J.W., Kim, H.S., & Park, N.G. Lewis Acid–base adduct approach for high efficiency perovskite solar cells. *Acc. Chem. Res.* **49**, 311-319 (2016).

2. Dualeh, A., Gao, P., Seok, S. I., Nazeeruddin, M. K. & Grätzel, M. Thermal behavior of methylammonium lead-trihalide perovskite photovoltaic light harvesters. *Chem. Mater*. **26**, 6160-6164 (2014).

3. Thanh, N. T. K., Maclean, N., & Mahiddine, S. Mechanisms of nucleation and growth of nanoparticles in solution. *Chem. Rev.* **114**, 7610-7630 (2014).
